# An Orbital Basis Set for Double Photoionization of Atoms and Molecules

**DOI:** 10.1021/acs.jctc.4c00929

**Published:** 2024-10-12

**Authors:** Roger Y. Bello, Frank L. Yip, Zachary Streeter, Robert Lucchese, C. William McCurdy

**Affiliations:** † Departamento de Química Física Aplicada, Módulo 14, Universidad Autónoma de Madrid, Madrid 28049, Spain; ‡ Department of Science and Mathematics, California State University Maritime Academy, Vallejo, California 94590, United States; § Department of Chemistry, University of California, Davis, California 95616, United States; ∥ Chemical Sciences Division, Lawrence Berkeley National Laboratory, Berkeley, California 94720, United States

## Abstract

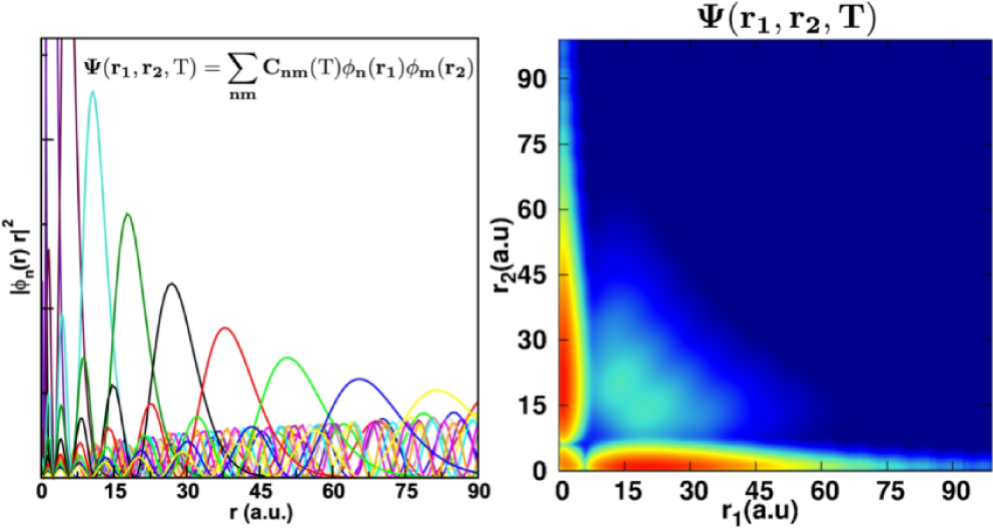

The *ab initio* theoretical treatment of one-photon double photoionization processes has been limited to atoms and diatomic molecules by the challenges posed by large grid-based representations of the double ionized continuum wave function. To provide a path for extensions to polyatomics, an energy-adapted orbital basis approach is demonstrated that reduces the dimensions of such representations and simultaneously allows larger time steps in time-dependent computational descriptions of double ionization. Additionally, an algorithm that exploits the diagonal nature of the two-electron integrals in the grid basis and dramatically accelerates the transformation between grid and orbital representations is presented. Excellent agreement between the present results and benchmark theoretical calculations is found for H^–^ and Be atoms, as well as the hydrogen molecule, including for the triply differential cross sections that relate the angular distribution and energy sharing of all of the particles in the molecular frame.

## 1 Introduction

The development of novel attosecond light sources has opened new possibilities for imaging and controlling electron dynamics in many-electron systems in their natural time scales. Notable examples include extracting photoionization time delays of molecules in the vicinity of Feshbach and shape resonances,^1−4^ monitoring the birth of a photoelectron,^5^ the observation of correlation-driven charge migration in a DNA building block,^6^ and retrieving real-space movies of the internal motion in molecules.^7,8^ Among the various light-induced phenomena, double photoionization (DPI) is one of the most fundamental processes. The photoelectrons ejection patterns provide a complete picture of the competition between the effects of electron–nuclear, and electron–electron interaction, as well as the acceleration of the electrons in the direction of the light polarization vector.^9^ In addition, due to its high sensitivity to electron correlation, DPI offers a unique insight into the nature of the collective electron dynamics of the target.

Complex computational methods to obtain DPI amplitudes have been developed in the last two decade. However, most of them have been exclusively directed to describing DPI in atoms and the H_2_ molecule. These methods work within the two-active electron approximation and usually make use of a FEM-DVR basis set to describe the two outgoing electrons.^9−11^ Other methods using Sturmian functions,^12,13^ Bsplines,^13,14^ or using hybrid basis e.g., combining Gaussian functions with FEM-DVR,^15,16^ and orbitals with FEM-DVR,^17−20^ have also been successfully employed.

Obtaining accurate DPI amplitudes in polyatomic molecules usually requires a significant increase in the size of the basis set. The size increase is 2-fold. First, higher angular momenta are needed to accurately describe the multicenter molecular potential, particularly the cusp at each nucleus. In addition, the number of angular configurations in the basis increases as the symmetry of the system decreases. Second, the density of the radial grid has to be increased in order to describe the highly compact core orbitals. This size increase makes the DPI problem almost computationally intractable, even for small polyatomic systems. In a recent work one-photon DPI amplitudes for H_2_O were reported, however these amplitudes were obtained averaging over all spatial orientations of the molecule and considering an independent-particle model for the molecular initial electronic state.^21,22^

In the present work, a novel energy-adapted orbital basis set implementation is described. The orbital basis effectively reduces the size of the basis without compromising the accuracy of the observables. The orbitals, which are eigenfunctions of the one-electron Hamiltonian, are used to describe the two photoelectrons, while the remaining core is kept frozen (the two active electron approximation). The energy gap between the valence and core electrons and the close-shell character of the core electrons make this approximation fairly accurate. Although we do not do so here, the frozen core approximation can in principle be lifted by coupling double ionization channels. The orbitals are expanded in a product of FEM-DVR functions and symmetry adapted spherical harmonics for the radial and angular coordinates, respectively.

The key feature of the orbital basis we construct here is that it can be energy adapted so as to reduce the size of the problem. The number of orbitals included in the wave function expansion is limited by the maximum energy of each electron. The energy threshold imposed not only reduces significantly the size of the basis but also eliminates (by construction) the high spectral terms in the Hamiltonian, allowing the use of larger time steps when solving the time-dependent Schrödinger equation (TDSE). All of the above advantages makes the orbital basis particularly well suited to describe DPI processes in small polyatomic molecules. As a demonstration of the orbital basis, we choose the relatively simple cases of DPI of H^–^ and Be atoms, and the H_2_ molecule. As theoretical data from previous works is available for the three systems,^12,14,17,18,23,24^ they represent an excellent testbed of the methodology presented here. We found an excellent agreement between the DPI amplitudes calculated with the present methodology and those reported in previous studies.

The outline of this paper is as follows. In Section 2 the theoretical framework for the orbital basis is discussed. The method to calculate the DPI amplitudes is detailed and some computational details are given. In Section 3 the DPI amplitudes calculated are compared with previous results obtained using different approaches. Finally in Section 4 we make some concluding remarks about the prospects for applying this approach to larger molecular targets.

## 2 Theory

Within two-active electron approximation the effective Hamiltonian for the two electrons can be written (atomic units will be used throughout):
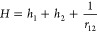
1where  is the Coulomb repulsion between the active electrons. The one-body operator *h* is
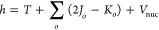
2where the sum is over occupied orbitals, *T* is the one-electron kinetic energy operator, *V*_nuc_ the nuclear attraction, and 2*J*_*o*_ and *K*_*o*_ are the direct and exchange components respectively of the closed-shell core interaction with the valence electrons. Explicitly, the Coulomb operator for the orbital  of symmetry Γ is given by
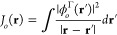
3The doubly occupied orbital is expanded in a product of FEM-DVR basis functions, , and symmetry adapted real spherical harmonics, , (see Appendix A) for radial and angular coordinates, respectively
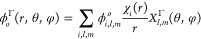
4with coefficients .

The matrix elements of the Coulomb operator in this basis is given by (dropping the symmetry symbol Γ for simplicity)
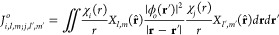
5in order to evaluate this six-dimensional integral we follow a procedure paralleling the computation of the pure FEM-DVR two-electron integrals. The strategy is to utilize a multipole expansion for the electron repulsion,
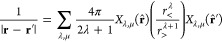
6Then, the radial integrals in the FEM-DVR basis can be reduced, using an approach that solves Poisson’s equation in the FEM-DVR basis, to an expression involving just the inverse of kinetic energy operator,^10^
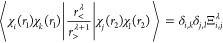
7where
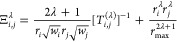
8and where  is the  element of the inverse of kinetic energy matrix for the λ angular momentum, *w*_*i*_ and *w*_*j*_ are the associated Gauss–Lobatto quadrature weights for FEM-DVR points *r*_*i*_ and *r*_*j*_, respectively. Importantly, the expression in eq 8 for the radial two-electron integrals is diagonal in the indices for each electron.

Using eqs 5–8 the matrix elements of the Coulomb operator can be written as
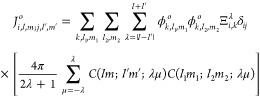
9where the angular integral,
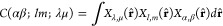
10is performed using a Lebedev–Laikov quadrature.^25^ Note that the  and  angular pairs are constrained by the symmetry Γ of the doubly occupied orbital ϕ_*o*_, while the  and  angular pairs are constrained by the total symmetry of the  matrix element.

The nonlocal exchange operator acting on an orbital  is given by
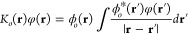
11Following the same steps taken to obtain the Coulomb operator matrix elements, the final expression for the exchange operator matrix elements can be written as,
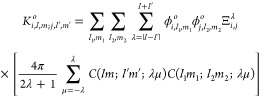
12

Finally, the electron–nucleus attraction potential is given by
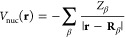
13where **R**_β_ and *Z*_β_ are the position and charge of the nucleus β, respectively. Using the same approach that is used to evaluate the Coulomb and exchange operators, the electron–nucleus potential matrix elements can be written as
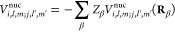
14where
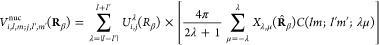
15and
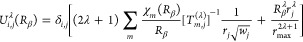
16

Note that calculating the electron–nucleus potential in the FEM-DVR basis involves evaluating the basis functions at the positions of the nuclei (see eq 16). Consequently, it is convenient to place one of the FEM-DVR boundaries at the nucleus position where such evaluation is straightforward .

### 2.1 Orbital Basis

The orbitals in the basis are chosen to be eigenfunctions of the one-body Hamiltonian in eq 2,
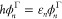
17where *n* is the index for a given eigenfunction of symmetry Γ and where the orbitals are written as linear combination of the grid basis set, similarly to the doubly occupied orbitals in eq 4
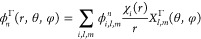
18

In subsequent equations we will drop the symmetry superscript Γ for the sake of simplifying the notation. Obtaining the two-electron integrals in the orbital basis  usually involves performing a four-index transformation of the two-electron integrals calculated in the underlying FEM-DVR basis.^26^ Here we avoid performing that transformation in its primitive form by taking an “electron-density” approach that exploits the underlying grid representation of the orbitals.

First, we take the product of the two orbitals with the same electron index,  and . This product is most efficiently obtained by transforming to the grid representation of the angular coordinates, in which we evaluate the values, , of the *nth* orbital at the angular point α and radial point *i*,
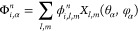
19where  are the Lebedev–Laikov quadrature points. Then, the density is obtained taking the product of the two orbitals in their grid representation. We note that in the two-electron integrals we only need terms in the density which are diagonal in the radial FEM-DVR functions. Thus, the density is given by
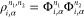
20This property of the two-electron integrals in the FEM-DVR basis is the one that is emulated by the tensor-hypercontraction approximation^27,28^ to two-electron integrals in a basis of Gaussian functions. The purpose of the tensor-hypercontraction approximation is to approach the much improved scaling with basis size that we describe below. Here the diagonal property of the two-electron integrals in eqs 7 and 8 is exact within the FEM-DVR quadrature and no further approximations are made.

Next, we transform the density, now in its grid representation, back to the partial wave representation. This transformation can be achieved by just integrating over the angular coordinates,^29^
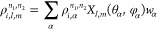
21where *w*_α_ is the Lebedev–Laikov quadrature weight associated with the  point. The electrostatic potential due to the density  can be written as
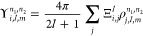
22where the radial two electron integrals, , are defined in eq 8. The final two-electron integrals in the orbital basis can then be obtained by taking the overlap of densities  and the  functions.
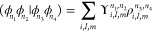
23

For *N* orbitals the number of two-electrons integrals is *N*^4^. This number can be significantly reduced taking into account the permutational symmetry between the orbitals^26^ in eqs 23. In addition, symmetry can be also exploited as the two-electron integral is different from zero only if the product of the point-group symmetry of each orbital is equal to the totally symmetric irreducible representation.

Finally, the matrix elements of the one-body Hamiltonians are easily constructed because the orbitals are chosen to be eigenstates of the one-body Hamiltonian,
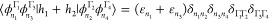
24

Employing time-independent orbitals provides the flexibility of only calculating the Hamiltonian matrix elements just a single time. Then, those matrix elements can be stored and used in different time-dependent calculations, e.g., for different pulse frequencies and time durations.

### 2.2 Double Ionization Amplitudes

The interaction of the target system with the radiation pulse is described by solving the time-dependent Schrödinger equation (TDSE),
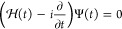
25where , with *H* being the system time-independent Hamiltonian in eq 1, and *V*_*t*_ is the laser-system interaction. Using the length gauge and within the dipole approximation the laser-system interaction is given by , where the electric field for a photon energy ω and total duration *T* can be written as
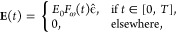
26where *E*_0_ is the maximum electric field amplitude and  is the light polarization direction. We have chosen a sine-squared envelope for the time dependence of the pulse ,
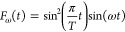
27

We solve eq 25 by expanding the time-dependent wave function in the orbital basis described above.
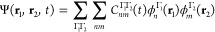
28The initial wave function at time *t* = 0, corresponding to the ground state of the system, is obtained by diagonalizing the time-independent Hamiltonian in eq 1. Since the ground state is either a singlet or a triplet,  is either an antisymmetric or symmetric function of **r**_1_ and **r**_2_, respectively. This permutational symmetry is then conserved at all times.

In order to extract the DPI amplitudes from the wave packet, we let it further propagate for an additional time *t*_*p*_ after the end of the pulse. Then, the double photoionization amplitudes are obtained by projecting the time-dependent wave function onto products of continuum “testing functions”  satisfying incoming boundary conditions,^30−34^
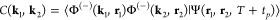
29Projecting the uncorrelated product of continuum wave functions onto the total time-dependent wave function has been used previously to extract double ionization amplitudes from wave packets for atoms and molecules to obtain results in excellent agreement with other extraction methods.^30−34^ The main limitation of this approach arises from the need to propagate the wave function for longer times than the pulse duration, but this method avoids the calculation of the surface integral expression for the double ionization amplitudes described by McCurdy et al.^10^

The functions  are the target cation continuum eigenfunctions with incoming momentum **k**. While there are other physically equivalent alternatives for testing functions, this choice is convenient since it eliminates the contributions of the single ionization channels to eq 29. The incoming continuum testing functions are related to the outgoing version by , and  satisfies
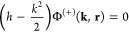
30We solve eq 30 by writing the  as a sum of Coulomb function and  and a scattered wave correction, ,
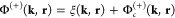
31The Coulomb wave function admits the partial wave expansion,
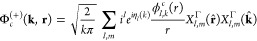
32where  is the radial Coulomb function that behaves asymptotically as , and  is the Coulomb phase. The scattered wave correction satisfies the driven equation,^10,14^
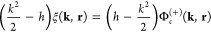
33Since ξ is an outgoing wave, the correct outgoing boundary conditions are imposed by solving eq 33 using exterior complex scaling.^10,35^

The fully differential cross section for a single photon double ionization process, can be formally written as,
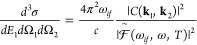
34where  is the Fourier transform of the pulse, and , with *E*_*i*_ and  being the ground state and final state energies, respectively. Resolving the continuum dynamics using the above expressions allows us, from a single time propagation, to extract fully differential cross sections for any given final energy *E*_*f*_ within the bandwidth of the pulse.

### 2.3 Computational Details

All the calculations were performed within the  point group symmetry. The basis set, associated with the expansion in eq 28, is energy adapted by including only the orbitals from eq 17 with an energy lower than the threshold energy of *e*_th_ = 1.1 au, 1.5 au, and 2.5 au for H^–^, Be and H_2_, respectively. We performed convergence studies (not shown here) that suggest that in general, for a given excess energy *E*_*f*_, convergence to graphical accuracy is reached by including all the orbitals with an energy such that , where *E*_ion_ is the energy of the cation ground state. Note that the value of the energy in parentheses here is the electron energy for a single ionization process. The size of the basis set is further reduced by restricting the value in the expansion of the wave function in eq 28 of the product  to the symmetry of the states involved in the process studied. For instance, for a single photon transition in the case of H^–^ and Be the pair , is restricted to values such that . We note that there is no formal limitation to use the orbital basis to study multiphoton double ionization processes, but that the symmetries accessible by each photon must be included.

Employing a radial basis of 265 FEM-DVR functions and  = 7 for the angular coordinates, in  symmetry, would produce a two-electron Hamiltonian of order . Setting the threshold energy in the orbital basis to *e*_th_ = 1.5 au results in a two-electron Hamiltonian of order , thus effectively reducing the order of the Hamiltonian by more than an order of magnitude.

The maximum single-electron angular momentum needed to converge the ground state energies and the triply differential cross sections (TDCS), in the energy range considered, was found to be  = 7 for the three systems studied. We note that the calculated double-ionization potentials are in very good agreement with previously reported values (see Table 1). The time dependent calculations were performed setting the pulse intensity and temporal duration to I = 3 W cm^–2^ and *T* = 0.5 fs, respectively. The wave packet was allowed to further propagate for an additional time of *t*_*p*_ = 1.0 fs after the end of the pulse. The time propagation was performed using a short-iterative Lanczos propagator^36,37^ with a time step of  a.u. Imposing an energy threshold for the orbitals included in the basis removes (by construction) the high energy eigenvalues of the one-body Hamiltonian, which in turn allows the use of larger time steps and more compact radial grids without altering any physical observable.

**Table 1:** Double Ionization Potentials for H^–^, Be, and H_2_^a^

System	Orb. (eV)	DVR (eV)	*Ex* (eV)
H^–^	14.30	14.36	14.36
Be	27.41	27.42	27.53
H_2_	51.23	51.37	51.39

a The present results are compared to results obtained using a FEM-DVR basis set and exact values. Exact energies, *Ex*, are from ref.^38^ for H_2_ (R = 1.4 a.u.), and from refs.^39^ and (40) for H^–^ and Be, respectively.

## 3 Results

### 3.1 H^–^ Double Photoionization

**Figure 1:**
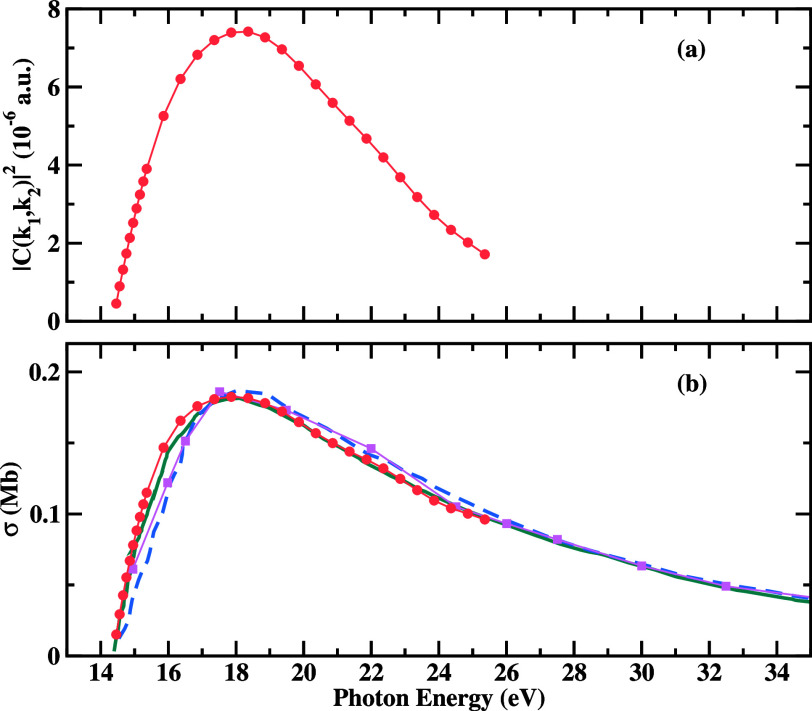
(a) Squared amplitudes integrated over the emission directions of the two electrons and the energy sharing between them, as a function of the photon energy. (b) Total single-photon double-ionization cross section of H^–^ as a function of the photon energy. Salmon solid circles: present results. Plum solid squares: results from ref.^12^. Blue solid line: results from ref.^23^. Dark-cyan dashed line: Results from ref.^24^.

Figure 1a depicts the absolute squared amplitudes  (see eq 29), for a central frequency =20 eV, integrated over the emission directions of the two electrons and the energy sharing between them, as a function of the photon energy. In this case the amplitudes for a given total electron kinetic energy and energy sharing were extracted by projecting the total wave function onto the product of two bare Coulomb functions with *Z* = 1 with the desired kinetic energies. The absolute square amplitudes given in Figure 1a reflect the bandwidth and central frequency of the attosecond pulse as well as the energy dependence total cross sections. The total cross section, presented in Figure 1b, is then obtained by dividing the absolute square amplitudes by the pulse Fourier transform. Our results are generally in good agreement with those reported previously including calculations obtained by wave packet propagation,^12^ convergent close coupling method,^23^ and a pure FEM-DVR basis set.^24^

**Figure 2:**
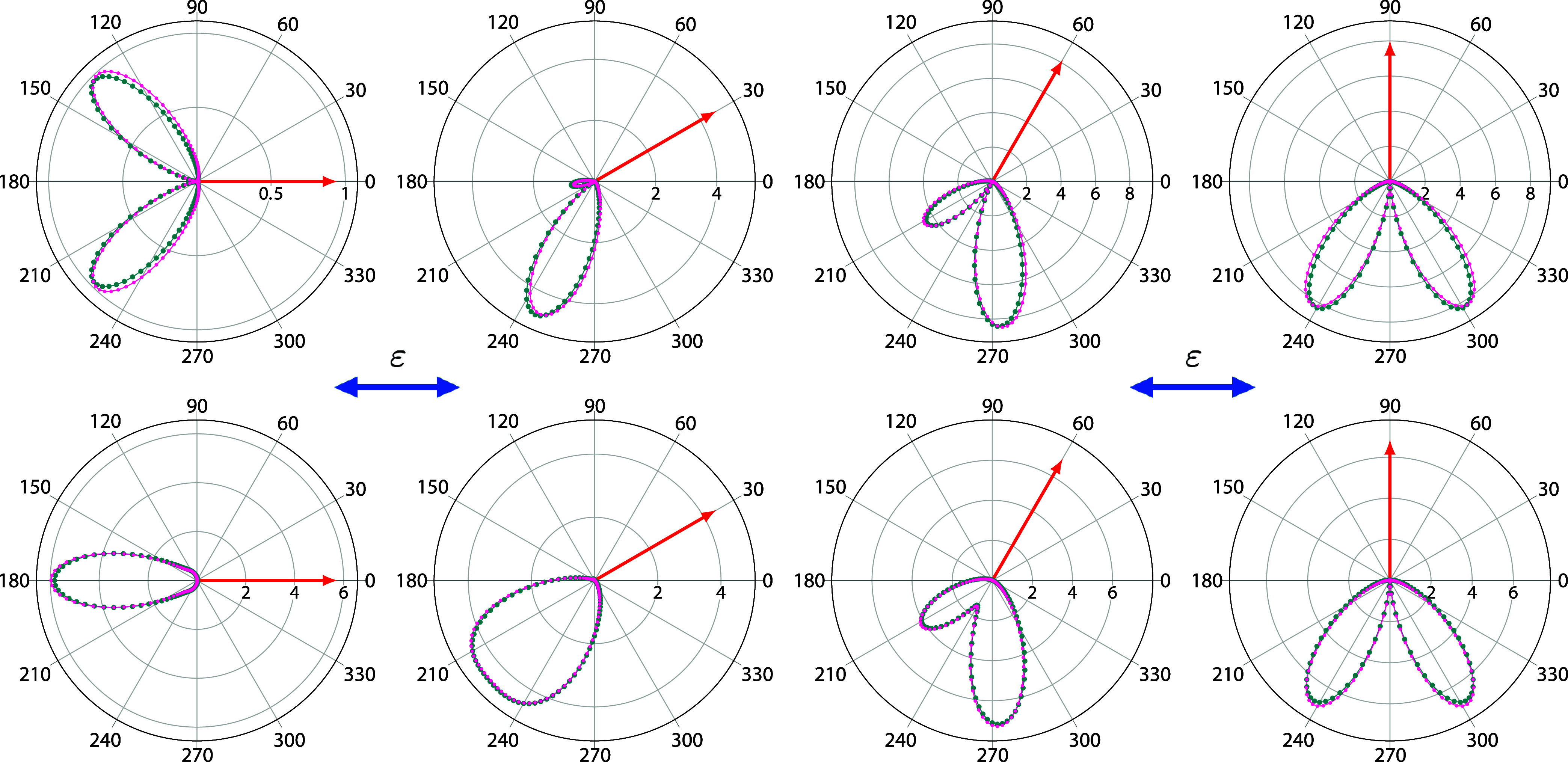
TDCS for double ionization of H^–^ at  =18 eV for in-plane geometries. Fixed electron (single ended red arrows) with 50 (upper row) and 90 (lower row) of the available energy and various directions with respect to the light polarization vector (double ended blue arrow). Dark cyan points: results from refs.^11^ and (24) (obtained using the velocity gauge). Magenta points: present results. Units are kbarn/eV/sr^2^.

A more robust test of the orbital basis is calculating the TDCS, which depends on the emission directions of the two electrons and on the energy sharing between them. The TDCS contains the signatures of the contributions of electron correlation to the dynamics. Thus, correlation in both the initial and final states must be properly treated to obtain accurate results.^11,24^ The TDCS for a photon energy of  = 18 eV (3.7 eV of excess energy) is presented in Figure 2, for various fixed-electron directions. The fixed electron carries away 50% (upper row) and 90% (lower row) of the total available energy. A comparison with converged benchmark calculations obtained using a pure FEM-DVR basis set^11,24^ is also presented. The agreement, both in magnitude and shape, between the present results and the corresponding FEM-DVR calculation is excellent.

In addition, both theoretical results exhibit the signature of parity-selection rules preventing both electrons from being ejected back-to-back at equal energy sharing, and preventing in general the emission of both electrons perpendicular to the light polarization direction.^41^ All of the above evidence indicates an accurate representation of the electron–electron interaction matrix elements encoding the physics that drives the double-ionization process.

### 3.2 Be Double Photoionization

**Figure 3:**
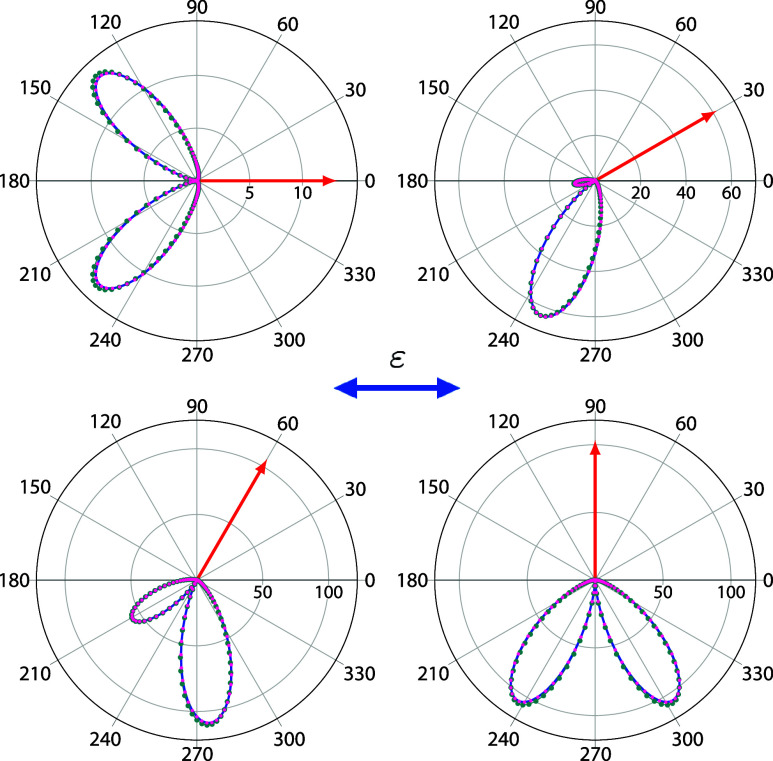
TDCS for double ionization of Be at  = 37.4 eV for in-plane geometries. Fixed electron (single ended red arrows) with 50 of the available energy and various directions with respect to the light polarization vector (double ended blue arrow). Dark cyan points: results from ref.^18^ (obtained using the velocity gauge). Magenta points: present results obtained using Coulomb functions as testing functions. Solid blue line: present results obtained using Be^+^ continuum states as testing functions. Units are kbarn/eV/sr^2^.

Figure 3 shows the TDCS for a photon energy of  = 37.4 eV (10 eV of excess energy), for various fixed-electron directions. The fixed electron carries away 50% of the total available energy. In this case, the TDCS were determined using a pulse of central frequency  = 40 eV. Then, the amplitudes in eq 29 were obtained using two different approaches. First, we projected the time dependent wave function onto the product of Be^+^ continuum eigenfunctions, calculated using Coulomb functions with *Z* = 2 (see eqs 30-33). Second, we neglected the short-range correction (see eq 31) in the testing function and projected the time-dependent wave function onto the product of two bare Coulomb functions. Using *Z* = 2 is a suitable choice since the long-range behavior of the direct operator for the () core () screens the *Z* = 4 nucleus of Be, and the exchange operator  has the range of the 1*s* orbital.

The TDCS in Figure 3 are compared with converged benchmark calculations obtained using a hybrid orbital-FEM-DVR basis set.^18^ The agreement, both in magnitude and shape, between the present results and the hybrid basis results is excellent. Both theoretical results exhibit the signature of parity-selection rules observed in Figure 2. In addition, the results obtained by projecting onto bare Coulomb functions and Be^+^ continuum states are graphically indistinguishable from each other. This means that, during the time propagation the wave packet has enough time to reach the asymptotic region, where the short-range correction is negligible and projecting onto the different testing functions should be equivalent. This serves as an additional test to the reliability of the extraction method in eq 29.

### 3.3 H_2_ Double Photoionization

**Figure 4:**
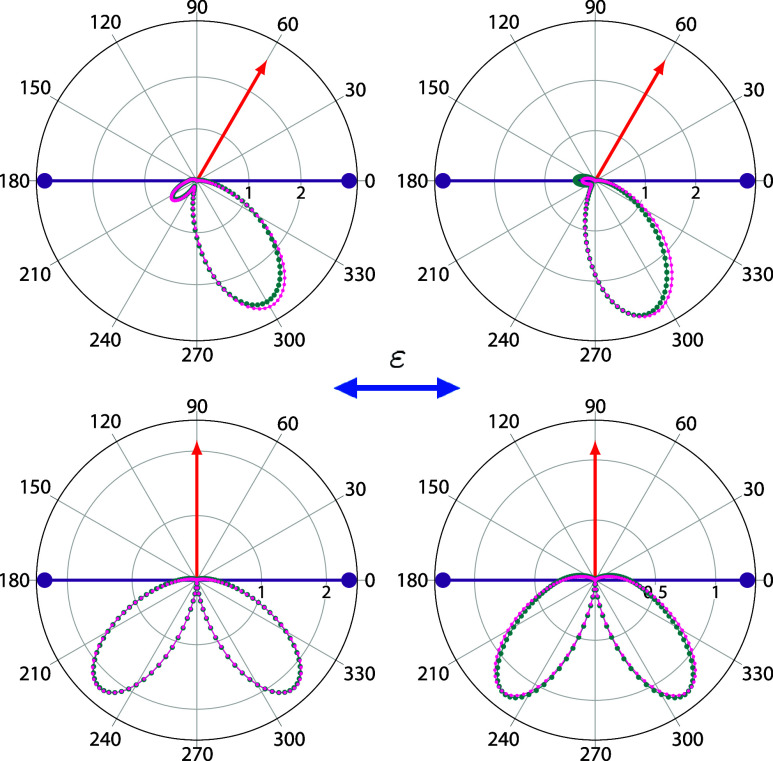
TDCS for double ionization of H_2_ at  = 61.4 eV for in-plane geometries. Molecule is oriented parallel to the light polarization vector (double ended blue arrow). Fixed electron (single ended red arrows) with 20% and 80% of the available energy and various directions with respect to the light polarization vector. Dark cyan points: FEM-DVR basis set (obtained using the velocity gauge). Magenta points: orbital basis set. Units are barn/eV/sr^2^.

**Figure 5:**
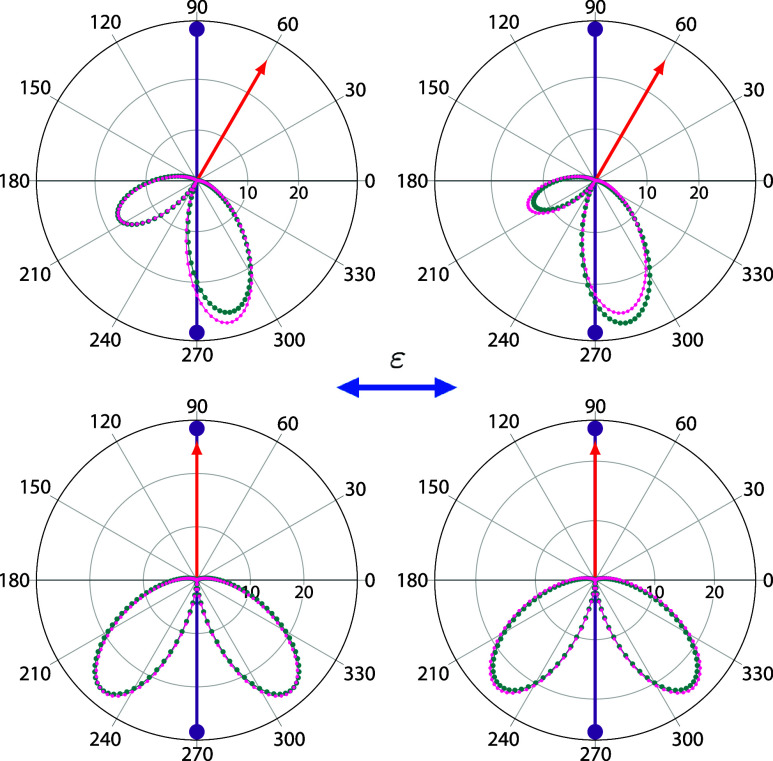
Same as Figure 4 but with the molecule oriented perpendicular to the light polarization vector. Units are barn/eV/sr^2^.

Our main motivation for developing an orbital basis method was to study double photoionization of molecular targets. TDCS in molecular targets are sensitive to electron correlation in both the initial, and final states.^11^ Thus, any comprehensive theoretical description of double ionization processes in molecular targets requires an accurate representation of the electron–electron interaction. In the present, we have chosen as benchmark system the H_2_ molecule which has been extensively studied both theoretically and experimentally.^9,11,14,32,42−45^Figure 4 shows the TDCS for a photon energy of  = 61.2 eV (10 eV of excess energy), for various fixed-electron directions with respect to the light polarization vector. The fixed electron carries away 20 (left column) and 80 (right column) of the total available energy. The light polarization vector is oriented parallel to the molecular axis, leading to the ^1^ (^1^) final symmetry. In this case, the TDCS were determined using a pulse of central frequency  = 62 eV, and projecting onto the product of H_2_^+^ continuum eigenfunctions, calculated using Coulomb functions with *Z* = 2. The corresponding TDCS for the light polarization vector oriented perpendicular to the molecular axis, i.e., ^1^ (^1^, ^1^) final symmetry, is presented in Figure 5. The TDCS are compared with converged benchmark calculations obtained using a pure FEM-DVR basis set. As in the previously examined cases of H^–^ and Be, the agreement between the present results and the FEM-DVR basis results is excellent. The small differences observed could be due to potential convergence issues that could be made visible by comparing the TDCS obtained using different gauges and is possibly magnified by the small magnitude of the cross section.

Although the ^1^ and the ^1^ cross sections differ in almost an order of magnitude, the TDCS for both molecular orientations (and each energy sharing), exhibit similar features, i.e., two lobes in the opposite direction from the fixed electron with no significant cross section in the back-to-back geometry. This feature can be characterized as atomic-like as they resemble the angular distributions obtained for H^–^ and Be for similar orientations of the fixed electron with respect to the polarization vector (see Figure 3).

## 4 Conclusions

In this work, we have developed and applied an energy-selected orbital basis set to describe DPI processes in atoms and molecules. A strategy for evaluating the relevant operator matrix elements has been given, including an efficient transformation between numerical grid and orbital basis representations. TDCS computed with the present method, for H^–^ and Be atoms, and for molecular hydrogen, and compared with benchmark theoretical calculations reveals an excellent agreement of the orbital basis results with the existing data. The results presented here provide confirmation of the present method for describing two electrons in the nontrivial molecular continuum, suggesting the utility of expanding this method for treating more complicated and experimentally relevant molecular targets in DPI studies.

Employing a single-centered basis set to describe DPI processes in polyatomic targets requires the use of high angular momenta in order to accurately describe the molecular potential. Thus, leading to a large number of orbitals in the basis. However, such calculations can be performed with the current implementation of this method for other linear or hydrogenated molecules, e.g., H_2_O, CH_4_, NH_3_, where the expansion center can be placed on the heavier atom. This potential issue could be circumvented by using a multicenter expansion placing a center on each atom.

## A Real Spherical Harmonics

Real spherical harmonics^46,47^ are defined as follow:

35

where

36

37
